# Hind feet position score: A novel trait to genetically reduce lameness incidence

**DOI:** 10.3168/jdsc.2023-0414

**Published:** 2023-10-06

**Authors:** A. Köck, J. Kofler, L. Lemmens, M. Suntinger, M. Gehringer, F.J. Auer, K. Linke, B. Riegler, C. Winckler, G. Berger, C. Egger-Danner

**Affiliations:** 1 ZuchtData EDV-Dienstleistungen GmbH, Dresdner Str. 89/18, 1200 Vienna, Austria; 2 Department of Farm Animals and Veterinary Public Health, University Clinic for Ruminants, University of Veterinary Medicine Vienna, Veterinärplatz 1, 1210 Vienna, Austria; 3 LKV-Austria, Dresdner Str. 89, 1200 Vienna, Austria; 4 Department of Sustainable Agricultural Systems, Institute of Livestock Sciences, University of Natural Resources and Life Sciences, Vienna, 1180 Vienna, Austria; 5 Rinderzucht Austria, Dresdner Str. 89, 1200 Vienna, Austria

## Abstract

Lameness is an important health and welfare issue that causes considerable economic losses in dairy herds. The objective of this study was to investigate whether the hind feet position score (HFPS) can be used as an auxiliary trait for genetic evaluation of lameness. The HFPS is evaluated by visual scoring of the position of both the hind-digits to the mid-line of the cow's body. The higher the heel height of the lateral claw, the higher is the HFPS, and the higher is the risk for development of lameness. In total, 3,478 records from 1,064 Fleckvieh cows from 35 farms were obtained between September 1, 2021, and March 5, 2022. Data collection was carried out by the regional milk recording organizations. Hind feet position was scored visually by trained personnel during routine milk performance testing in the milking parlor using a 3-class scoring system: score 1 = 0° to <17° indicating a balanced heel height of both the medial and the lateral claw; score 2 = angle of 17° to 24°; score 3 = angle of >24°. After all cows had been milked, locomotion scoring was performed for each animal using a 5-class scoring system with locomotion scores ranging between 1 (normal) and 5 (severely lame). Using HFPS, sensitivity and specificity were 69.5% and 66.8%, respectively, for detecting lameness defined by locomotion score ≥2. For genetic analyses, a bivariate linear animal model was fitted with fixed effects of herd, parity, lactation stage, and classifier, and random effects of animal and permanent environment. Heritabilities for HFPS and locomotion score were 0.07 and 0.10, respectively, and the genetic correlation between the 2 traits studied was 0.80. These results suggest that the HFPS could be used for genetic evaluations to reduce lameness incidence in dairy cattle.

## Highlights

• Hind feet position score was evaluated in the milking parlor during routine milk performance testing and compared with outcomes from gait scoring.
• Hind feet position score is a heritable trait and shows a high genetic correlation to the locomotion score.
• At the phenotypic level, hind feet position score is of limited value to identify individual lame cows.

## Graphical Abstract

**Figure:**
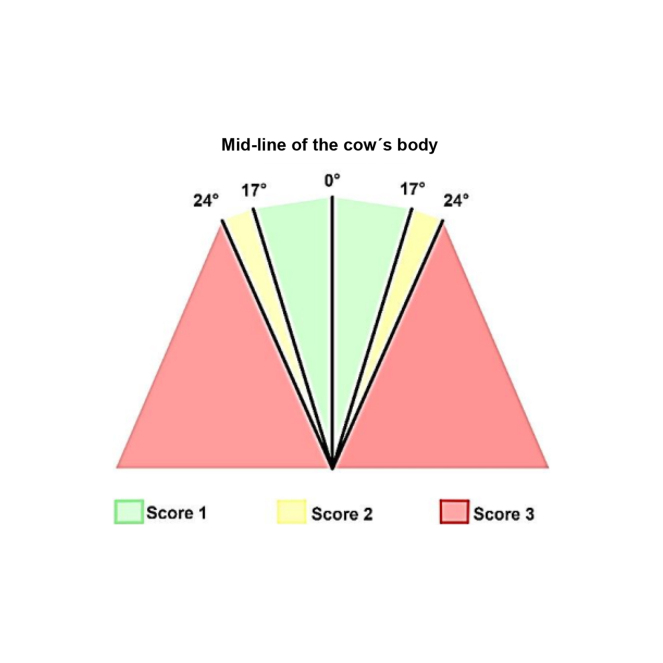
**Summary:** This study investigated the extent to which the assessment of hind feet position score, recorded during routine milk performance testing in the milking parlor, can be used as an auxiliary trait for genetic evaluation of lameness in dairy cows. For hind feet position scoring, sensitivity and specificity were 69.5% and 66.8%, respectively, for detecting lameness defined by a locomotion score ≥2. Genetic parameters for hind feet position score and locomotion score were estimated. The heritability for hind feet position score was 0.07 and the locomotion score was 0.10. The genetic correlation between the 2 traits was 0.80. These results suggest that the value for hind feet position score could be used for genetic evaluations to reduce the incidence of lameness in dairy cows.

Lameness is a severe welfare problem in dairy cattle and indicates pain or discomfort during locomotion. Lameness is defined as an abnormal stance or gait of the animal that results from disorders of the locomotor system (Sprecher et al., 1997). In most cases, lameness is caused by disorders of the claws or the distal legs and reflects the animal's attempt to reduce the load on the affected and painful limb (Murray et al., 1996). In dairy cows, lameness is one of the most common health disorders, along with fertility problems and mastitis, with mean prevalence ranging from 14.9% to 50.4% in Austria (Burgstaller et al., 2016; Kofler et al., 2022; Lemmens et al., 2023). Lameness seriously impairs animal welfare due to its association with pain as well as reduced eating time and activity (Norring et al., 2014; Weigele et al., 2018; Lemmens et al., 2023), resulting in lower milk, fat, and protein production (Kofler et al., 2021); impaired fertility (Fürst-Waltl et al., 2021; Logroño et al., 2021); increased susceptibility to metabolic disorders (Calderon and Cook, 2011); higher culling rates (Puerto et al., 2021; Kofler et al., 2022); and costs for increased labor and treatment (Ózsvári, 2017; Robcis et al., 2023).

Locomotion scoring according to Sprecher et al. (1997) is a frequently used method worldwide to identify lameness, especially in research studies (ICAR, 2022). This method uses a 5-point scale to evaluate the gait pattern of cows in freestalls or on pasture. It focuses on the detection of unloading movements of the limbs and the evaluation of the backline (flat or arched) both at rest and during walking. The main problem in detecting early lameness stages in cows is that observation of gait is time consuming and not used routinely by farmers in Austria. An alternative to locomotion scoring in the moving animal would be to observe standing cows for indicators associated with lameness during milking. Zurbrigg et al. (2005) used the arched back and the outward rotation of hind claws as indicators for assessment of lameness in tiestalls. Leach et al. (2009) developed a stall lameness score for detecting lameness in cows in tiestalls using indicators or behaviors such as weight shifting, rotation of feet, standing on the edge of a step, resting a foot, and uneven weight-bearing.

Bulgarelli-Jiménez et al. (1996) introduced the hind feet position scoring system to provide a method for detecting lameness that does not require the presence of any of the characteristic signs described by many authors used to detect lameness (Sprecher et al., 1997; Hoffman et al., 2014; ICAR, 2022; Werema et al., 2022). This method of hind feet position scoring visually determines the degree of external rotation of the hind feet using a 3-class scoring system. It was developed to assist decision-making regarding the optimal time for functional hoof trimming in dairy herds. A few months after hoof trimming the outer hind claw is commonly distinctly higher than the inner claw, resulting in claw overload and stimulation of horn growth (van der Tol et al., 2004; Sadiq et al., 2020; Fischer et al., 2021). These conditions lead to an increased pressure on the sole horn and therefore also on the dermis, and therefore increase the risk for sole hemorrhages, double soles, sole ulcers, and white line lesions causing a painful sensation (Machado et al., 2010; Griffiths et al., 2020).

Since locomotion scoring using the method of Sprecher et al. (1997) is not routinely recorded in Austria by milk recording employees during regular milk performance testing or by stockpersons, a method for assessing lameness in the milking parlor during routine milk performance testing was explored. For this purpose, the hind feet position score (**HFPS**) was selected.

The objective of this study was to evaluate the feasibility of observing the HFPS during milking and to compare the HFPS results with those of locomotion scoring. Phenotypic and genetic parameters were estimated to investigate the use of HFPS as an auxiliary trait for locomotion scoring in genetic evaluations.

Data collection was carried out by the regional milk recording organizations from September 1, 2021, to March 5, 2022, on 37 selected farms. Hind feet position scoring of dairy cows was performed by trained personnel every 4 to 6 wk during the course of routine milk performance testing. Hind feet position was scored on the standing cows in the milking parlor using a 3-class scoring system (Bulgarelli-Jiménez et al., 1996; Holzhauer et al., 2005; Reiter, 2022; Lemmens et al., 2023). The HFPS is evaluated by visual scoring of the position of both the hind-digits (angle formed by the line of interdigital space of each claw) to the mid-line of the cow's body (the line along the vertebral column). Scoring is done by a visual assessment from the back while the cows stand still. The angle (0° to >24°) is caused by differing heel heights of medial and lateral claws on each limb—the higher the heel height of the lateral claw, the higher the score. Physiologically the angle formed by the interdigital line and the body-midline ranges between 0° and <17° (score 1) indicating a balanced heel height of both the medial and the lateral claw. The height of both the heels are approximately equal here, thus there is no overload of one claw. A score 2 describes an angle of 17° to 24° and score 3 an angle of >24°. All cows with a score of 2 and especially a score of 3 are assumed to have a higher risk of developing pressure-related claw horn disruption lesions and should therefore be subjected timely to functional hoof trimming. Such preventive hoof trimming should equalize the height difference and reduce excessive pressure on one claw (usually on the outer claw) to prevent the occurrence of lameness (Bulgarelli-Jiménez et al., 1996; Holzhauer et al., 2005).

After all cows had been milked, locomotion scoring of each animal was performed in the alleys according to the locomotion scoring system of Sprecher et al. (1997), with 1 = normal (not lame), 2 = slightly lame, 3 = moderately lame, 4 = lame, and 5 = severely lame.

Analyses were carried out only for Fleckvieh cows, which is the main breed in Austria. After edits, 3,478 records from 1,064 cows from 35 dairy farms were available for analyses. An animal pedigree file was generated by tracing the pedigrees of cows back as far as possible (in total 23,897 animals).

Hind feet position scores 2 and 3 as a predictor of lameness, defined as locomotion score ≥2 (Sprecher et al., 1997), were evaluated in terms of sensitivity, defined as the proportion of lameness cases correctly classified, and specificity, defined as the proportion of non-lame cows correctly classified.

Phenotypic and genetic parameters were estimated with a bivariate linear animal model using the average information-restricted maximum likelihood (AI-REML) procedure in the DMU package (Madsen and Jensen, 2008). The following model was applied to all traits:

**y** = **Xβ** + **Z_pe_pe** + **Z_a_a** + **e**,

where **y** is a vector of observations (locomotion score and HFPS); **β** is a vector of systematic effects, including fixed effects of herd, parity, lactation stage, and classifier; **pe** is a vector of random permanent environmental effects; **a** is a vector of random animal additive genetic effects; **e** is a vector of random residuals; and **X**, **Z_pe_**, and **Z_a_** are the corresponding incidence matrices.

Locomotion scores were considered as class variable with 1, 2, 3, 4, and 5. The same was applied for HFPS with classes 1, 2, and 3. Parity had 5 classes with 1, 2, 3, 4, and 5+. Lactation stage was defined in 4 classes (1 ≤ 90 DIM, 2 = 91–180 DIM, 3 = 181–270 DIM, 4 >270 DIM).

The frequency of lameness cases was 31.6% (locomotion score ≥2), while 11.6% of lameness cases were moderate to severe (locomotion score ≥3). Frequency of records with a higher degree of external rotation of hind feet (HFPS ≥2) was 44.7%. Lameness frequency based on locomotion scoring was lowest in first-parity cows (18.2%) and increased with parity number (45.9% in fifth and higher parity; Figure 1). Higher parity was also associated with increased frequency of higher degree of external rotation of hind feet (HFPS 2 and 3; Figure 2). Parity order is a known risk factor for lameness caused especially by claw horn disruption lesions (Randall et al., 2018; Bran et al., 2019; Sahar et al., 2022). In comparison to primiparous cows, second, third, and fourth and higher parity cows have 1.6, 3.3, and 4 times higher odds of being lame, respectively (Solano et al., 2015; Kofler et al., 2021). For lameness, the effect of parity may be due in part to the increased likelihood of recurrence, as previous cases are a risk for future cases. Randall et al. (2018) estimated that between 79% and 83% of lameness events were due to exposure to all previous lameness events and between 9% and 21% were due to exposure to lameness events that occurred at least 16 wk previously. This could either be because certain cows are initially susceptible and remain susceptible, due to the increased risk associated with previous lameness events, or due to interactions with environmental factors (Randall et al., 2018).

**Figure 1:**
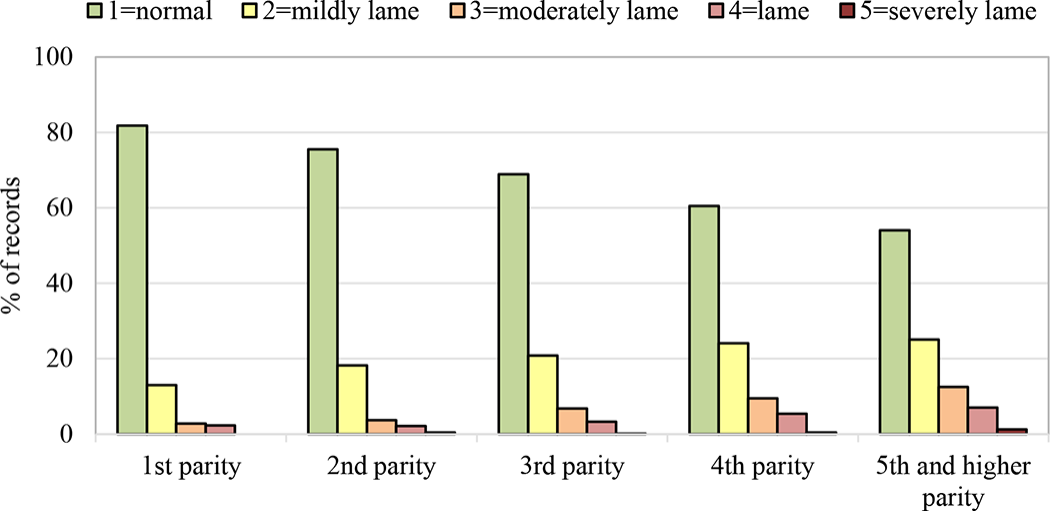
Percentage of locomotion records with score 1 (not lame) to 5 (severely lame) by parity.

**Figure 2:**
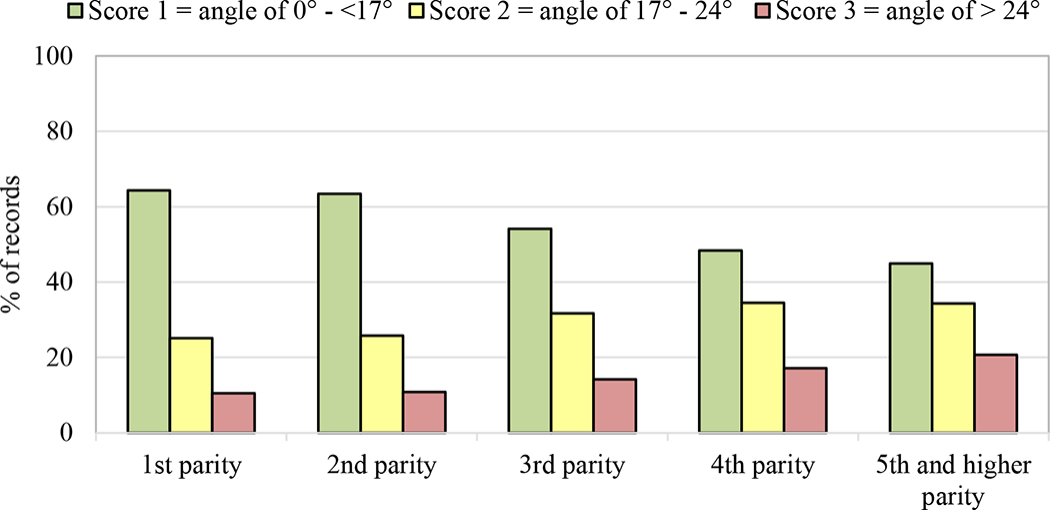
Percentage of hind feet position records with score 1, 2, and 3 by parity.

For detecting lameness (locomotion score ≥2) by HFPS, sensitivity and specificity were 69.5% and 66.8%, respectively. Leach et al. (2009) reported a sensitivity ranging from 0.54 to 0.77 for their stall assessment protocol based on multiple lameness indicators, depending on observer and threshold definition. Hoffman et al. (2014) examined the relationship of individual lameness indicators, like presence or absence of an arched back, cow-hocked posture, and preference for one leg when standing, with locomotion scores. All these indicator traits have been associated with locomotion scores but have not been highly sensitive or specific as diagnostic tests. For detecting moderate and severe lameness (locomotion score ≥3), sensitivity and specificity were 0.63 and 0.64 for arched back, 0.54 and 0.57 for cow-hocked (hocks were rotated medially), and 0.05 and 0.98 for favored-limb as they continued to stand (Hoffman et al., 2014). However, they concluded that the observation of these indicators may be useful as a screening test to identify cows that need further examination for lameness. Riegler (2022) examined indicators of lameness in standing cows. Regarding the detection of animals which had been classified as severely lame by gait scoring, the trait indicators of lameness while standing had a high specificity (88%–96%), but the sensitivity was low (30%–45%; i.e., the proportion of those animals that were identified as lame while standing and simultaneously classified as highly lame with the mobility score was low). Recently, Werema et al. (2022) compared lameness indicators assessed in the milking parlor (shifting weight, abnormal weight distribution, swollen heel or hock joint, and overgrown hoof) with locomotion scores. Using the lameness indicators assessed in the milking parlor, a decision tree machine learning procedure classified cows with locomotion score class ≥2 with a true positive rate of 75% and a false positive rate of 0.2%. They concluded that in-parlor scoring using defined lameness indicator traits has the potential to be an alternative to locomotion scoring on pasture-based dairy farms.

The phenotypic correlation between HFPS and locomotion score was positive, but low (0.38; Table 1). In agreement with our results, Lemmens et al. (2023) found a correlation of 0.39 between HFPS and locomotion score based on a different data set that included farms with freestall barns with an automatic milking system.

**Table 1:** Heritability (on the diagonal), genetic correlation (above the diagonal), and phenotypic correlation (below the diagonal) with SE in parentheses for hind feet position score and locomotion score

Item	Hind feet position score	Locomotion score
Hind feet position score	**0.071 (0.036)**	0.80 (0.27)
Locomotion score	0.38	**0.096 (0.039)**

Heritability estimates for HFPS and locomotion score were 0.07 and 0.10, respectively (Table 1). Similarly, Onyiro and Brotherstone (2008) found a heritability of 0.11 for locomotion measured according to the linear type classification guideline of Holstein cows. Weber et al. (2013) reported a heritability of 0.08 for locomotion defined as a binary trait (0 = locomotion score 1 or 2, 1 = locomotion score ≥3). Köck et al. (2018) found a heritability of 0.07 for locomotion score.

In the present study, a high positive genetic correlation of 0.80 was found between HFPS and locomotion score (Table 1). Although the estimate was associated with a rather high standard error, the result was significantly different from zero. We could not find any results in the literature on genetic correlations between HFPS and locomotion score. This is probably due to the fact that the main intention of the HFPS system is to identify a difference in heel height, especially in cows that do not yet show any lameness and should be preventively hoof trimmed.

In conclusion, our results suggest that lame animals are not sufficiently reliably detected using HFPS. However, the estimated genetic parameters indicate that HFPS could be used for genetic evaluations to reduce lameness incidence in dairy cows. While interobserver reliability should be further studied, the advantage of this new trait for identifying lameness is that it can be recorded in the milking parlor (e.g., during routine milk performance testing but also regularly by the farmers themselves after prior training).
